# Noncoding RNAs, Emerging Regulators of Skeletal Muscle Development and Diseases

**DOI:** 10.1155/2015/676575

**Published:** 2015-07-14

**Authors:** Mao Nie, Zhong-Liang Deng, Jianming Liu, Da-Zhi Wang

**Affiliations:** ^1^Department of Orthopaedic Surgery, The Second Affiliated Hospital, Chongqing Medical University, 76 Linjiang Road, Chongqing 400010, China; ^2^Department of Cardiology, Boston Children's Hospital, Harvard Medical School, 320 Longwood Avenue, Boston, MA 02115, USA

## Abstract

A healthy and independent life requires skeletal muscles to maintain optimal function throughout the lifespan, which is in turn dependent on efficient activation of processes that regulate muscle development, homeostasis, and metabolism. Thus, identifying mechanisms that modulate these processes is of crucial priority. Noncoding RNAs (ncRNAs), including microRNAs (miRNAs) and long noncoding RNAs (lncRNAs), have emerged as a class of previously unrecognized transcripts whose importance in a wide range of biological processes and human disease is only starting to be appreciated. In this review, we summarize the roles of recently identified miRNAs and lncRNAs during skeletal muscle development and pathophysiology. We also discuss several molecular mechanisms of these noncoding RNAs. Undoubtedly, further systematic understanding of these noncoding RNAs' functions and mechanisms will not only greatly expand our knowledge of basic skeletal muscle biology, but also significantly facilitate the development of therapies for various muscle diseases, such as muscular dystrophies, cachexia, and sarcopenia.

## 1. Introduction

One of the biggest surprises from the human genome project is that, in contrast to large predicted gene numbers, our genome contains only 20,000~25,000 protein-coding genes, which account for merely ~1.5% of the whole genome [1]. Over the last decade, it has been gradually accepted that the remaining genomic information, originally considered “noise” or “dark matter,” is not “junk DNA” after all. Through comprehensive analyses of mammalian transcriptomes, a vast amount of non-protein-coding RNAs (ncRNAs), including microRNAs (miRNAs) and long noncoding RNAs (lncRNAs), have recently been identified. They are emerging as integral components of the gene regulatory networks in a broad range of biological processes, and dysregulation of their expression has been implicated in many human diseases [2, 3]. miRNAs are a class of small noncoding RNAs (approximately 22 nucleotides long) that are evolutionarily conserved from plants to mammals. Generally, miRNAs negatively regulate their targets at the posttranscriptional level by promoting mRNA degradation and/or repressing translation [2]. On the other hand, lncRNAs are transcripts normally longer than 200 nucleotides in length, which do not appear to have protein-coding potential [3]. Only recently discovered, they represent a new class of ncRNAs that have also been implicated in a large spectrum of biological processes.

Skeletal muscle consists of about one-third of our body mass and is the largest tissue in our body. Attached to bones through tendons, skeletal muscle is not only responsible for generating voluntary movement, but also very adaptive and actively participates in metabolism of the whole body. Skeletal muscle is also known for its remarkable ability to regenerate after injury through the use of satellite cells, the endogenous muscle stem cells [4–6]. Satellite cells usually stay quiescent in resting muscle; however, once activated, they can reenter the cell cycle, proliferate, and initiate the myogenic differentiation program. Over the past few decades, a wealth of knowledge has been accumulated regarding the molecular regulatory networks of muscle development and pathophysiology [4, 7, 8]. However, we are still at the beginning of understanding the roles of ncRNAs in skeletal muscle biology. Although aberrant expression of both miRNAs and lncRNAs has been associated with various muscle disorders, such as muscular dystrophies, the functions and mechanisms of these ncRNAs remain unclear [9–12]. Therefore, understanding the functions of miRNAs and lncRNAs during skeletal muscle development and under pathophysiological conditions will greatly expedite the development of therapeutic treatments for many muscle disorders. Here, we briefly summarize the known functions of miRNAs and lncRNAs in skeletal muscle development and pathophysiology.

## 2. miRNAs, Small Yet Mighty Regulators

### 2.1. miRNA Biogenesis and Function

More than 1,000 miRNAs are encoded in the human genome. They are abundantly expressed in many cell types and are estimated to target roughly 60% of mammalian genes [2]. miRNAs are either transcribed independently from their own transcriptional units or cotranscribed with the host genes in which they are embedded. The biogenesis pathway of miRNAs has been well documented and is evolutionarily conserved. Briefly, immature primary transcripts containing a stem-loop structure (pri-miRNAs) are initially transcribed by RNA polymerase II [13]. Pri-miRNAs are processed by the Drosha/DGCR8 endonuclease complex into ~70 nucleotides precursor miRNAs (pre-miRNAs) inside the nucleus. The pre-miRNAs are then exported to the cytoplasm, where they are further processed by the Dicer/TRBP endonuclease complex, resulting in imperfect RNA duplexes [14–16]. The mature miRNA duplexes are then separated and incorporated into the RNA-induced silencing complex (RISC), where they bind to the 3′ untranslated regions (UTRs) of their target mRNAs [17, 18]. Thus, miRNAs contribute to posttranscriptional regulation of gene expression by translational inhibition and/or target mRNA degradation. Generally, nucleotides 2–8 of miRNAs, termed the “seed sequence,” are essential for target specificity and binding. Many miRNAs are clustered into families based on their seed sequence; thus, miRNAs with the same seed sequence may target the same set of genes, providing the possibility of functional redundancy and cooperation among different miRNAs [19–21].

miRNA-mediated gene regulation is a complex and well-orchestrated process. Unlike transcriptional factor-mediated regulation of gene expression, which appears to be “on or off,” miRNAs tend to only moderately regulate the overall level of their target's expression, so they are referred to as “fine-tuners” [21]. Although the effect of a single miRNA on a specific gene may appear to be small, the combinatory effect of miRNAs on multiple mRNA targets functioning within the same biological pathway can be synergistic and sometimes dramatic [22, 23]. In addition, a mRNA normally possesses multiple miRNA-binding sites in its 3′ UTR and is likely a target for numerous different miRNAs. Therefore, this reciprocal multiplicity between miRNAs and mRNAs increases not only the complexity but also the robustness of the miRNA regulatory network. This, to some extent, may also explain why knockouts of many miRNAs, when deleted individually in animal models, do not result in apparent defects.

However, it should be noted that this moderate regulation of gene expression does not necessary suggest that miRNAs are dispensable. Perhaps the most convincing evidence supporting the importance of miRNAs in mammals is the embryonic lethal phenotype of Dicer knockout mice [24]. Dicer is an endonuclease, encoded at a single locus in mammals, which is required for generating biologically active mature miRNAs. Dicer-deficient mice die at the gastrulation stage before the embryo body is fully developed, demonstrating that functionally mature miRNAs are critical for early mammalian development [24]. More specifically, the importance of miRNAs in skeletal muscle has also been demonstrated through a Dicer conditional loss-of-function study in skeletal muscle. Using a MyoD-Cre recombinase transgene to remove a conditional Dicer allele in skeletal muscle, Harfe and colleagues found that collective loss of functional miRNAs in skeletal muscle resulted in muscle hypoplasia, increased apoptosis, and perinatal lethality [25], clearly demonstrating the critical role of miRNAs in skeletal muscle. Here we will discuss the function of some known miRNAs in skeletal muscle development (myogenic differentiation), muscle physiology (fiber types, hypertrophy/atrophy), and the pathophysiology of muscle diseases.

### 2.2. miRNA in Skeletal Muscle Development (Myogenic Differentiation)

Since the first report of a myogenic regulatory miRNA by our and other groups [26–28], increasing numbers of miRNAs have been identified as able to modulate myogenesis. In Table 1, we list the miRNAs that have been implicated in myogenesis, along with their identified target(s). Due to space limitations, here we will only summarize some well-characterized miRNAs with validated targets in myogenic differentiation.

**Figure 1:**
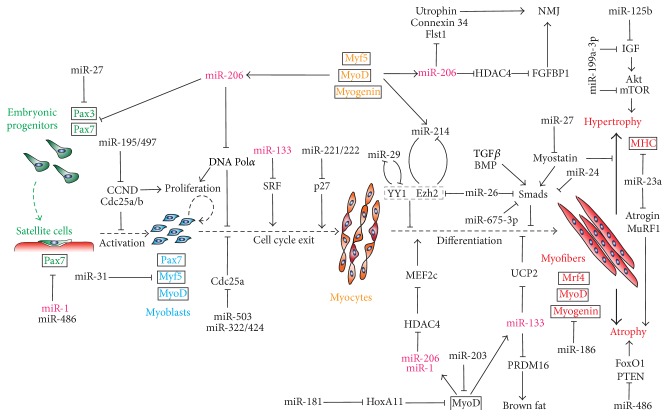
miRNAs in myogenesis. The diagram shows various miRNAs and targets that regulate the quiescence and activation of satellite cells, proliferation of myoblasts, and their subsequent cell cycle exit and terminal differentiation into myofibers. The myogenic transcription factors play a central role by governing the expression of several muscle-enriched miRNAs such as miR-1, miR-206, and miR-133 (magenta). The myogenic transcription factors characteristic of each stage of the myogenic process are marked in corresponding colors in a black box. The listed targets include paired box genes 3 and 7 (Pax3 and Pax7), serum response factor (SRF), DNA polymerase catalytic subunit (DNA pol*α*), cell-division cycle protein 25A (Cdc25A), homeobox A11 (HoxA11), Yin and Yang 1 (YY1), enhancer of zeste homolog 2 (Ezh2), histone deacetylase 4 (HDAC4), insulin-like growth factor (IGF), uncoupling protein 2 (UCP2), and PR domain containing 16 (PRDM16).

Myogenesis is a complex process that requires coordination of multiple factors governing activation of quiescent satellite cells, proliferation of myoblasts, cell cycle exit, and subsequent terminal differentiation resulting in multinucleated myofibers [4, 5]. Over the past few decades, using C2C12 cells as an* in vitro* model of myogenesis, together with numerous* in vivo* studies, the regulatory networks of these factors have been well defined [4, 7, 8, 29]. It is now known that both adult muscle regeneration and embryonic myogenesis share a strikingly similar genetic hierarchy orchestrated by a cascade of myogenic transcription factors, including the paired box (PAX) family of transcription factors (Pax3/7), the basic helix-loop-helix (bHLH) myogenic regulatory factors (MRFs) such as Myf5, MyoD, myogenin, and MRF4, and MEF2 and SRF [4, 7, 8, 29]. These transcription factors can act in either synergy or antagonism through feedforward and feedback loops. Recently, miRNAs have been shown to play critical roles in myogenesis through their reciprocal regulatory relationship with these transcription factors (Figure 1). miRNAs can target several myogenic transcription factors, thus affecting myogenesis; on the other hand, their own expression is sometimes directly controlled by these transcription factors [10, 30]. This intricate relationship between miRNAs and myogenesis regulatory factors supports a view of miRNAs as an integral part of the regulatory network of myogenesis (Figure 1). Indeed, several miRNAs have been identified as directly suppressing myogenic transcription factors and are thus able to control the progression of myogenic differentiation. For example, miR-186 targets myogenin and thus inhibits terminal muscle differentiation [31]. Similarly, miR-181 promotes MyoD activity by repressing its negative regulator HoxA11 in myogenic cells [32]. It could be envisioned that further studies will identify novel miRNAs controlling myogenesis by directly regulating myogenic transcription factors.

Satellite cells are muscle stem cells that have the capacity to differentiate into muscle fibers upon muscle damage, yet maintain a quiescent status under normal conditions. Two paired box transcription factors, Pax3 and Pax7, are essential for maintaining this muscle stem cell status through their antidifferentiation capacity, owing to their negative regulation of MyoD [4, 6, 8]. Others and we have found that the miR-1/206 family, comprised of miR-1-1, miR-1-2, and miR-206, is capable of promoting myogenesis, in part by inhibiting Pax3/7 in embryonic muscle precursors and satellite cells [33–35]. The miR-1/206 family is transcribed from three different chromosomal loci in the form of bicistronic transcripts with miRNAs in the miR-133 family (miR-133a-1, miR-133a-2, and miR-133b) [28, 33, 36]. miR-1-1 and miR-1-2 are expressed in both skeletal and cardiac muscles, while miR-206 is specific for skeletal muscle [28, 30, 33, 36]. Similarly, miR-27b has been found to regulate Pax3 levels in embryonic myotomes and activate satellite cells to stimulate myogenic robustness during embryonic myogenesis [37]. In addition, miR-486 can target the Pax7 3′ UTR in satellite cells [38]. Therefore, most miRNAs modulate satellite cells' quiescent status and/or myogenic activation by directly targeting Pax3/7, the master transcription factors that maintain satellite cell quiescence. Alternatively, the recently identified function of miR-31 represents a new mode of miRNA action for regulating satellite cell myogenic activation. miR-31 helps satellite cells maintain their quiescence state by sequestering Myf5 mRNA in mRNP granules in the cell nucleus. Upon satellite cell activation, downregulation of miR-31 in these cells results in rapid release of Myf5 mRNA for protein translation and subsequent myogenesis initiation [39].

Upon activation, Pax3/7 expression is immediately reduced, while several bHLH myogenic transcription factors such as Myf5 and MyoD are upregulated in satellite cells [8]. During embryonic myogenesis or muscle regeneration, activated myoblasts first undergo a quick phase of active proliferation that normally produces an ample number of myoblasts before they exit the cell cycle and start terminal differentiation. Interference with either the proliferation or cell cycle exit stage of activated myoblasts will result in premature or delayed differentiation. Therefore, in theory, any miRNA that affects cell proliferation or the cell cycle will be able to control myogenic differentiation. In myoblasts, several highly expressed miRNAs appear to coordinately regulate this process. Activated by MyoD in activated myoblasts, miR-206 not only can repress Pax3/7 expression, thus further stimulating myoblast activation, but also can repress the p180 subunit of DNA polymerase alpha, whose downregulation coincides with cell cycle exit and differentiation of various tissues [40, 41]. Interestingly, although miR-133 is also upregulated during C2C12 differentiation and muscle regeneration, it promotes myoblast proliferation by inhibiting serum responding factor (SRF) [26]. Since SRF is directly involved in the regulation of miR-1/miR-133a transcription [26, 28, 42], miR-133 and SRF form a negative feedback circuit that balances myoblast proliferation and differentiation. In addition, several other miRNAs (miR-195/497, miR-322/424, and miR-503) have been recently identified as capable of regulating myoblast proliferation by targeting Cdc25a/b and CCNDs, all well-known cell cycle regulators [43, 44].

Once myoblasts exit the cell cycle, they become committed myocytes. MyoD and myogenin then coordinately drive their terminal differentiation by activating muscle specific transcription that was originally suppressed by the polycomb group (PcG) comlpex [4, 6, 8]. Enhancer of zeste homolog 2 (Ezh2) is a histone lysine methyltransferase subunit of the PcG complex that mediates MyoD- and myogenin-dependent promoter silencing in myoblasts through H3K4 and H3K27 methylation [45, 46]. Thus, downregulation of the PcG complex, allowing subsequent MyoD binding to activate muscle genes, is required for myoblasts to appropriately progress into myogenic differentiation. Interestingly, upregulation of several miRNAs (miR-214, miR-26, and miR-29) collaboratively represses PcG complex expression and function in myocytes and thereby promotes muscle-specific gene expression and differentiation (Figure 1). In undifferentiated myoblasts, PcG occupies a MyoD enhancer site upstream of miR-214, which is released by MyoD upon differentiation, allowing the activation of miR-214 expression. Therefore, by targeting and inhibiting Ezh2, miR-214 forms a negative feedback loop with the PcG complex [47]. Similarly, miR-26a also targets and suppresses Ezh2 in differentiating myocytes, although it is not clear if MyoD regulates its expression [48, 49]. Like miR-214, miR-29 forms a negative feedback loop with several other critical components of the PcG complex such as Yin Yang 1 (YY1), RING, and Rybp, an YY1 binding protein, by inhibiting their expression [50, 51]. Furthermore, miR-29 can also regulate myogenesis more directly by repressing AKT3 in differentiating myocytes [52].

While both the miR-1/miR-206 family and miR-133 family of miRNAs become enriched in myocytes during differentiation, likely via MyoD- and/or myogenin-dependent transcriptional regulation, their effects and mode of action on myogenesis are different. miR-1/206 inhibits the expression of histone deacetylase 4 (HDAC4), which suppresses the MEF2c transcriptional activity to induce muscle gene expression. Thus, this forms a positive feedforward loop in which MEF2c induces miR-1 expression, causing inhibition of the MEF2c repressor HDAC4, leading to enhancement of MEF2 activity [26]. In contrast, miR-133 inhibits myogenic differentiation and sustains myoblast proliferation by inhibiting SRF, as mentioned above. Since miR-133 has been recently implicated in the regulation of brown adipose differentiation by directly targeting PRDM16, a master transcription factor for brown adipogenesis [53], and there are common Myf5 positive progenitor cells for brown fat and skeletal muscle during embryonic development, miR-133 likely participates in regulating the adipogenic/myogenic fate determination in such progenitor cells [54]. In fact, miR-133 was recently found to control the brown adipose fate determination of satellite cells [55]. This is particularly intriguing and warrants future investigation into miR-133's functions in myogenesis and adipogenesis to elucidate how this single miRNA can regulate the cell fate determination and switching.

During terminal muscle differentiation, numerous cell signals can affect the activity of myogenic transcription factors, thus fine-tuning and adjusting the outcome of myogenic differentiation [6, 8]. Therefore, miRNAs that regulate these signaling pathways will also profoundly affect the outcome of myogenic differentiation. For example, myostatin, a member of the transforming growth factor *β* (TGF*β*) family, has been implicated in the negative regulation of muscle growth and regeneration [56]. Recently, miR-27a/b was shown to promote myogenic differentiation by targeting the 3′ UTR of myostatin, in addition to inhibiting Pax3 levels during embryonic myogenesis [57, 58]. Inhibiting miR-27a/b by antagomir led to increased myostatin levels and decreased myoblast proliferation and satellite cell activation, whereas miR-27a/b overexpression can reduce myostatin levels and induce skeletal muscle hypertrophy [57, 58]. Likewise, TGF-*β*s and BMPs both inhibit myogenesis through the activation of Smads [56]. Many miRNAs are found to regulate Smad levels in myocytes, thus affecting the robustness of myogenesis. For example, miR-24 can promote myogenic differentiation by targeting and inhibiting Smad 3 [59], while miR-26a represses Smad 1 and Smad 4 expressions [49]. miR-675-3p is another recently identified miRNA that promotes muscle differentiation by targeting Smads 1 and 5; interestingly, it is encoded in the exon of a long noncoding RNA, H19, which also plays a critical role in myogenesis [60].

As the final step of myogenesis, newly formed multinuclear myotubes will undergo further maturation processes that eventually result in functional myofibers with specialized structures such as neuromuscular junctions (NMJs). Interestingly, miRNAs have also been implicated in the maturation of muscle fibers. For example, miR-206 is enriched in neuromuscular junctions (NMJs), where it plays important roles in promoting the formation of NMJ endplates [61]. This function of miR-206 is mediated, at least in part, by its target, HDAC4, which inhibits the expression and release of FGFBP1 [61]. Since increased FGF pathway activity is required for NMJ reformation during muscle fiber maturation, removing its repressor HDAC4 locally at NMJs by miR-206 is critical for forming functional skeletal muscle [61]. Consistent with its NMJ-specific role, miR-206 deregulation has been associated with the adult motor neuron disease Amyotrophic Lateral Sclerosis (ALS), also known as Lou Gehrig's disease and myotonic dystrophy type 1 (DM1) [61, 62]. Lastly, miR-206 has been found to regulate various other muscle-specific genes also important for the maturation of skeletal muscle, including Connexin 43 (CX43), Follistatin-like 1 (Fstl1), and Utrophin (Utrn) [41, 63, 64].

In summary, as myogenesis progresses from myogenic precursors or satellite cells to functional myofiber formation, miRNAs participate in virtually every step of the way, tightly integrate into the myogenic regulatory network. As the miRNA field enters a more mature stage, more and more muscle-specific mechanisms of miRNA regulation will certainly be discovered.

### 2.3. miRNAs in Skeletal Muscle Physiology (Fiber Type and Hypertrophy/Atrophy)

All mammals contain a variety of skeletal muscle subtypes differing in their origin and physiological properties [8]. One of the physiological characteristics that reflect muscle heterogeneity is the difference in distinct fiber types between various muscle groups [65]. Conventionally, muscle fibers have been classified as type I (slow) and type II (fast) fibers, where type II fibers consist of three subtypes, IIa, IIb, and IIX. The main difference among these fiber types is in the composition of myosin heavy chain (MyHC) isoforms [65]. Separate genes located at different genomic loci encode these MyHC isoforms in humans [65]. Thus, fiber type determination was traditionally thought to be regulated predominantly at the transcription level [66]. However, this view has been recently challenged by the identification of a group of miRNAs called MyomiRs. These intronic miRNAs, miR-208a, miR-208b, and miR-499, are embedded in three muscle-specific MyHC genes (*Myh6, Myh7, and Myh7b,* resp.) [67, 68]. Among these three miRNAs, miR-208a is specifically expressed only in cardiac muscle, whereas miR-208b and miR-499 are also expressed in type I (slow) muscle fibers [67].

These MyomiRs play important roles in regulating muscle myosin isoforms, thus regulating muscle fiber type. The two MyomiRs miR-208b and miR-499 have redundant functions in controlling muscle fiber type by repressing fast myofiber genes while activating slow muscle specific genes [67, 68]. Overexpression of miR-499 in skeletal muscle completely converts the fast myofibers of the soleus muscle into slow fibers [67]. Conversely, double knockout of miR-499 and miR-208b in mouse leads to a dramatic loss of type I fibers in the soleus muscle [67]. The effects of MyomiRs on muscle fiber identities are largely mediated by targeting transcriptional repressors of slow muscle fiber genes, such as Sox6, Pur*β*, and Sp3 [67]. Interestingly, knockout of Sox6 in skeletal muscle in mice indeed results in a fast-to-slow myofiber conversion [69–71]. It is noteworthy that MyomiRs are all embedded in the introns of various muscle myosin heavy chain genes, thus sharing the same expression levels as their host genes [72]. On the other hand, the expression of these MyHC genes is in turn regulated by the transcription factors targeted by these MyomiRs. Therefore, it appears that nature has utilized an endogenous feedback mechanism between MyomiRs and myosin heavy chains for fine-tuning muscle fiber type determination in order to modulate muscle physiology and performance.

Skeletal muscle not only has a remarkable regenerative capacity but also is a very adaptive tissue. Exercise improves muscle function, enhances muscle energy metabolism, and leads to muscle hypertrophy, which is characterized by increasing muscle mass [73, 74]. On the contrary, immobilization, lack of exercise, or other diseases, such as cancer, often induce muscle atrophy, which is defined as loss of muscle mass and reduced metabolic activity. Besides changes in the expression and activity of many muscle enzymes and proteins, the expression of many miRNAs is also altered during muscle hypertrophy or atrophy [75–77]. For example, miR-23a and miR-696 are increased in muscle hypertrophy after endurance exercise and conversely decreased in muscle atrophy induced by muscle immobilization [77–79]. Both of these miRNAs can negatively regulate peroxisome proliferator-activated receptor gamma coactivator 1-*α* (PGC-1*α*), a key regulator of metabolism and mitochondria biosynthesis in skeletal muscles [78, 79]. In addition, miR-23a can regulate Atrogin and MuRF1, two E3 ubiquitin ligases important for degradation of muscle sarcomeric proteins during muscle atrophy [80]. Another miRNA participating in muscle atrophy regulation is miR-486, which inactivates atrophy signaling in skeletal muscle through combinatory inhibition of its targets PTEN and FoxO1 [81].

Physiological muscle atrophy (sarcopenia) also occurs with aging. Several recent studies profiling miRNA expression in skeletal muscle of young and old mice have revealed that several miRNAs, including miR-206, miR-698, miR-744-5p, and miR-468, are increased, whereas others, such as miR-29, miR-434, miR-455, miR-382, miR-181a, and miR-221, are reduced in skeletal muscle cells of old animals [82–84]. While the exact targets and molecular mechanisms of most of these differentially expressed miRNAs are still elusive, it is likely that they play important roles in skeletal muscle by coordinating with regulatory proteins of muscle atrophy.

### 2.4. miRNAs in Skeletal Muscle Disease Pathophysiology

Skeletal muscle diseases are a large group of heterogeneous muscle disorders that are characterized, at least in part, by muscle wasting. Most studies on miRNA function in muscle diseases have been focused on identifying dysregulated miRNAs using genome-wide analyses, such as microarrays or next-generation RNA deep sequencing. For example, work from Kunkel's lab has profiled miRNA expression in 10 major muscular disorders in humans and identified 185 miRNAs that are significantly up- or downregulated in pathological conditions. Of these, miR-146b, miR-155, miR-214, miR-221, and miR-222 are of special interest, as their expression is altered in almost all of the muscle diseases studied [85]. With miRNA profiling data becoming increasingly available, more and more miRNAs are implicated in the physiopathology of various muscle diseases. Here we provide a brief discussion on some of the most well-studied miRNAs in muscle pathophysiology.

The most common skeletal muscle disorder is muscular dystrophy, a large group of over 30 inherited muscle diseases characterized by progressive muscle wasting accompanied by repeated muscle degeneration and regeneration [86, 87]. The most common and severe muscular dystrophy is Duchenne muscular dystrophy (DMD), which is caused by mutations in the X-linked dystrophin gene [88]. Loss of dystrophin at sarcolemma in skeletal muscle results in fragility of myofibers, leading to muscle degeneration, chronic inflammatory responses, and fibrotic and fatty tissue deposition. Currently, there is no effective therapy for DMD. In the skeletal muscle of DMD patients, muscle tissues undergo profound cellular and molecular changes. Several microarray studies have been conducted using muscle tissues from either DMD patient or the* mdx* mouse, an animal model for human DMD [85]. Many MyomiRs and muscle-enriched miRNAs, such as miR-1, miR-133, and miR-206, are all increased in the serum of DMD patients and/or in muscle tissues of* mdx* mice [89–96]. This is consistent with their known role in myogenesis, since there is constant muscle degeneration and regeneration in dystrophic muscle, which requires the upregulation of these miRNAs to direct appropriate myogenic differentiation. Although there are still some inconsistences between various microarray studies, several miRNAs have been consistently identified to be dysregulated and to play a role in the pathophysiology of dystrophic muscles.

One of such miRNAs is miR-486, whose expression is downregulated in the muscle of DMD patients and* mdx* mice [85]. Using miR-486 transgenic mice, Alexander and colleagues demonstrate that overexpression of miR-486 alleviates the dystrophic phenotype of* mdx* mice, likely by regulating dedicator of cytokinesis 3 (DOCK3), platelet-derived growth factor receptor *β* (PDGF-*β*), and the PTEN/AKT pathway, thus affecting the cell cycle and muscle regeneration in* mdx* muscle [97]. Another downregulated miRNA in dystrophic muscles is miR-29, which positively regulates myogenic differentiation and reduces fibrosis. Consistent with its function, overexpression of miR-29 in* mdx* mice improves muscle regeneration, accompanied by reduced fibrosis [98]. In contrast, miR-199-5a is increased in* mdx* muscle [85]. miR-199-5a regulates the levels of several Wnt signaling pathway proteins, including Frizzled 4 (FZD4), Jagged1 (JAG1), and WNT2 [99]. All of these signaling proteins collectively regulate myoblast proliferation and differentiation; therefore, blocking this signaling pathway through excessive miR-199-5a in dystrophic muscle would likely affect myogenesis and muscle regeneration [99]. Of particular interest, miR-31 is also increased in dystrophic muscles. Besides enlisting a special mechanism to keep satellite cells in a quiescent state [39], miR-31 also directly targets the 3′ UTR of dystrophin transcripts [100]. Thus, miR-31 plays a dual role in dystrophic muscle: preventing satellite cell activation on one hand and promoting degradation of dystrophin mRNA on the other.

Studies using mice lacking both miR-133a-1 and miR-133a-2 (miR-133 dKO) reveal an important role of miR-133a in muscle pathophysiology [101]. In adult skeletal muscle of miR-133 dKO mice, a higher proportion of central nuclei was observed in type II (fast) muscle fibers, reminiscent of human central nuclear myopathy (CNM), a group of congenital myopathies characterized by abnormally centrally localized nuclei and muscle weakness [101]. Mutations of the dynamin 2 gene in human have been associated with CNMs in a dominant negative fashion, and increasing the level of dynamin 2 also leads to CNMs [102, 103]. Interestingly, miR-133a targets the 3′ UTR of dynamin 2 to repress its protein level. Therefore, the central nuclear myopathy observed in miR-133a dKO mice is at least partially due to the elevated protein level of dynamin 2 [101].

Rhabdomyosarcomas (RMS) are the only muscle cancer in humans, and they are the most common soft tissue sarcomas in children [104, 105]. One of the defining characteristics of RMS is the overexpression of myogenic differentiation markers, such as MyoD and Desmin [104, 105]. It has been reported that miR-1 and miR-206 levels are repressed in RMS [106], likely resulting in the inhibition of terminal differentiation of myogenic progenitor cells. Conversely, overexpression of miR-206 in RMS cells promotes their terminal differentiation and blocks tumor growth, thus highlighting the important role of miR-206 in RMS pathogenesis. One miR-206 target, the MET tyrosine-kinase receptor (MET) oncogene, may underlie such functions of miR-206 in RMS [107, 108]. Nevertheless, further studies are needed to definitively establish the role of miR-206 in RMS.

Over the past few years, miRNAs have been implicated in many aspects of muscle diseases. Owing to its molecular nature, small, abundant, and stable, miRNA can be easily detected. Indeed, several studies have attempted to use miRNAs as biomarkers in diagnosing specific forms of muscle diseases [62, 89, 90, 109]. Remarkably, several miRNAs, including MyomiRs and muscle-enriched miRNAs, have been detected in the serum of either DMD patients or* mdx* mice [62, 90, 91, 109]. The ability to detect such miRNAs in the serum of patients will provide valuable biomarkers and open up opportunities for developing miRNA-based diagnostic assays. Most importantly, given their important roles in skeletal muscle, miRNAs hold great promise for developing effective therapeutic interventions for a wide range of muscle diseases.

## 3. lncRNAs, Novel Regulators of Muscle Biology

### 3.1. lncRNAs, Definition and Classification

Although lncRNAs were only recently discovered, it has become increasingly clear that this class of noncoding RNAs regulates a variety of biological responses and that they do so via multiple different mechanisms [134]. So far, more than 10,000 lncRNAs have been identified in humans, and the number is likely to continue growing [135]. Present in both the nucleus and cytosol, lncRNAs are probably the most functionally diverse RNAs; they can act at nearly all steps of gene expression within the cell. This is largely due to the intrinsic nature of RNA molecules and their ability to form complex secondary structures, which enable them to bind to a diverse set of molecules, such as DNA, RNA, and proteins. Most lncRNAs are transcribed by RNA polymerase II and share mRNA-like features such as the 5′ cap, polyA tail, and splicing sites. In general, the transcription level of lncRNAs is lower compared to that of mRNAs, and transcription is cell context-dependent [136].

Based on their genomic locations and contexts, lncRNAs can be classified into several types: (1) intergenic lncRNAs (lincRNAs); (2) intronic lncRNAs; (3) sense-overlapping lncRNAs, and (4) antisense lncRNAs [3, 137]. Intergenic lncRNAs are long noncoding RNAs located between annotated protein-coding genes, generally in close proximity to neighboring protein-coding genes. In contrast, both sense and antisense lncRNAs at least partially overlap with exon sequences of annotated protein-coding genes and only differ from each other by their transcriptional directions. As the name suggests, an intronic lncRNA is an lncRNA that overlaps with the intronic region of a coding gene, and its transcription can be in either the sense or antisense direction.

**Figure 2:**
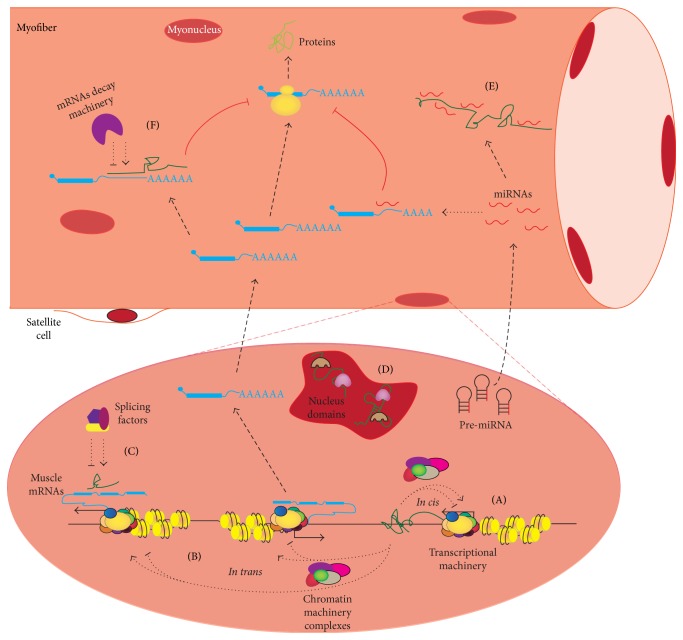
Functional mechanism of lncRNAs in skeletal muscle. (A) lncRNAs (in green) are able to positively or negatively regulate transcription at their own loci and at neighboring genes* in cis* by modulating transcriptional machinery or recruiting chromatin modification complexes. (B) Similarly, lncRNAs can also regulate a wide range of muscle gene expression* in trans* by recruiting chromatin modification enzymes and transcription machinery to their promoters. (C) lncRNAs can also regulate muscle gene mRNA splicing events by enlisting various splicing factors. (D) lncRNAs can serve as scaffolding for nuclear domains and/or long range chromosomal looping. (E) In cytosol, lncRNAs can serve as a miRNA sponge by competitively sequestering certain miRNA, thus inhibiting their effects on gene expression. (F) Recently, both SINE containing lncRNAs and H19 have been found to regulate mRNA decay by distinct mechanisms, therefore affecting muscle mRNA stability and regulating myogenesis.

Functionally, lncRNAs can also be divided into several subcategories according to their mode of action. (1) Many nucleus-enriched lncRNAs can exert their functions at the transcriptional level, either through cotranscriptional interactions between the nascent lncRNAs and transcriptional complexes or by the recruitment of such complexes like the chromatin modification enzymes, to transcription sites* in cis* or* in trans*. The* in cis* nature of an lncRNA refers to its ability to act on a neighboring gene on the same allele from which itself is transcribed; thus, this type of lncRNA commonly forms a feedback loop for regulation of itself and its neighboring genes. Still, many lncRNAs are found to also function in a* trans* mode to target gene loci distant from where the lncRNAs are transcribed. (2) Many lncRNAs can act as decoys for certain molecules, such as transcriptional/splicing factors in the nucleus and miRNAs or RNA degradation complexes in cytoplasm, to regulate the expression of targets of those biological pathways. (3) Furthermore, lncRNAs can also serve as a scaffold for forming complex molecular machineries or nuclear subdomains, profoundly affecting the expression level of many genes under various biological contexts. Clearly, additional modes of action for lncRNAs will likely be proposed as more functional lncRNAs are discovered. Nevertheless, example lncRNAs for all of the above-mentioned mechanisms have been identified in skeletal muscle development or pathophysiology, supporting the notion that lncRNAs are a class of evolutionary conserved molecules that affect a wide spread of biological processes (Figure 2).

Despite the rich information that we have learned about the transcription network regulating muscle development in the last few decades, we are still at the beginning of an “era of noncoding RNA.” Emerging literatures regarding several newly identified lncRNA functions in skeletal muscle have demonstrated that lncRNAs indeed are another integral part of the regulatory network of muscle biology. Clearly, lncRNAs have emerged as important novel regulators of skeletal muscle biology and diseases.

### 3.2. lncRNAs in Transcriptional Regulation of Skeletal Muscle Genes

Recently, a large number of lncRNAs were identified as being transcribed from enhancer regions of genes. These chromatin loci are usually occupied by transcriptional coactivator p300/CBP and RNA polymerase II and marked with histone H3K4 monomethyl (H3K4me1) and H3K27 acetyl (H3K27ac) modifications [138, 139]. These enhancer RNAs can regulate chromosome structure or transcription machinery through either a* cis-* or* trans*-mediated mechanism [140–147]. In two enhancer regions of the* MyoD* gene, the Distal Regulatory Regions (DRR) and the Core Enhancer (CE), two enhancer-associated lncRNAs, ^DRR^RNA and ^CE^RNA, were recently identified through a series of ChIP-seq experiments [148]. It has been proposed that ^CE^RNA facilitates the occupancy of RNA Pol II* in cis* by increasing chromatin accessibility, stimulating the expression of* MyoD*, while ^DRR^RNA functions* in trans* to promote the expression of myogenin, a key member of the myogenic transcription factor family [148]. More recently, Mueller and colleagues also identified an lncRNA transcribed upstream of* MyoD*, named MUNC (MyoD upstream noncoding RNA), and demonstrated that one of the spliced isoforms of MUNC is indeed ^DRR^RNA. Consistent with the previous study, experimentally decreasing MUNC expression blocked myoblast differentiation, further highlighting the role of these enhancer-associated lncRNAs during myogenesis [149]. Interestingly, these enhancer RNAs (eRNAs) seem to provide a positive feedback mechanism to reinforce the myogenic differentiation commitment upon satellite cell activation. Additional studies, in particular genetic studies in animal models, will be required to further establish the function of these lncRNAs* in vivo*. It will also be interesting to determine whether the expression and function of these lncRNAs are associated with any muscle diseases.

Similarly, a ChIP-seq study of Yin Yang 1 (YY1), an important component of the PcG complex that negatively regulates myogenesis, identified a number of lncRNAs regulated by YY1 (YY1-associated muscle lncRNAs or Yams) [150]. Individual Yams show distinct expression patterns during myogenesis and affect muscle differentiation differently; while Yam-2 and Yam-3 can promote muscle differentiation, Yam-1 and Yam-4 are negative regulators of myogenesis. Among the Yams, Yam-1, a single exon lncRNA, is the best studied. Like YY1, Yam-1 is downregulated during myoblast differentiation and during muscle development. Knockdown of Yam-1 can overcome YY1-mediated inhibitory effects on myogenesis, suggesting that Yam-1 is an important mediator of YY1 during skeletal muscle differentiation [150]. Interestingly, Yam-1 displays a* cis* effect on the expression of neighboring genes, one of which encodes miR-715, which targets and represses Wnt7b in skeletal muscle [150]. Thus it is also likely that some of the Yam-1 myogenic inhibitory effects can be attributed to* in cis* activation of miR-715, which in turn downregulates Wnt7b.

Another example of an* in cis* regulatory lncRNA is Glt2/Meg3, an lncRNA originating from the complex, imprinted* Dlk1-Dio3* region of the human genome [151, 152]. Containing protein-coding RNAs, lncRNAs, miRNAs, and snoRNAs, the imprinted* Dlk1-Dio3* locus has a complicated transcriptional regulation mechanism [151, 152]. The Glt2/Meg3 lncRNA is found to bind and recruit Polycomb Repressive Complex 2 (PRC2) to repress the transcription of* Dlk1* and Glt2-as, another lncRNA encoded by the* Dlk1-Dio3* region [152]. Knockout of Glt2/Meg3 results in perinatal lethality with defects in skeletal muscle development, highlighting its critical role in myogenesis [153]. It remains to be determined whether Glt2/Meg3 can also function* in trans* to regulate the expression and function of additional genes in skeletal muscle.

Besides regulating chromatin accessibility, some lncRNAs can also directly regulate specific transcription factors to modulate gene expression during myogenesis. Steroid receptor RNA activator (SRA) is such an lncRNA. Enriched in myofibers, it can selectively enhance transcriptional activation through steroid receptors [154, 155]. As a matter of fact, SRA lncRNA was the first lncRNA found to regulate myogenic differentiation [154, 156]. During myogenic differentiation, SRA lncRNA forms a functional complex with p68/p72, a member of the DEAD-box family of RNA helicases, and MyoD to facilitate chromatin remodeling and the formation of the transcription initiation complex at a subset of muscle-specific genes [154, 156]. Remarkably, the SRA transcripts also undergo alternative splicing and give rise to an mRNA encoding the protein SRAP, an RNA binding protein that specifically binds to SRA lncRNA and antagonizes its interaction with MyoD and p68/p72 [156, 157]. Interestingly, the ratio of the protein-coding and noncoding isoforms of the SRA transcripts changes during muscle differentiation. Thus, SRA lncRNA and protein form a reciprocal negative feedback loop that fine-tunes MyoD activity during myogenic differentiation.

### 3.3. lncRNAs as Molecular Decoys to Regulate Muscle Differentiation

lncRNA H19, the first lncRNA identified in mammals, originates from the imprinted* H19/IGF2* region [158]. Transcribed from the maternal allele, the H19 lncRNA is highly expressed in embryos as well as in adult skeletal muscle, suggesting its conserved function in regulating myogenesis [60, 159, 160]. MyoD directly activates H19 lncRNA transcription by interacting with a mesodermal enhancer of the* H19/Igf2* locus. H19 in turn downregulates* IGF2* expression* in trans* by recruiting the PRC2 repressor complex during muscle differentiation [161–163]. In addition, the H19 lncRNA contains several binding sites for the let-7 family of miRNAs, thus functioning as a molecular sponge/decoy for the major miRNAs of the let-7 family, which play critical roles in a wide range of physiological and pathological processes, including myogenesis [164]. Indeed, overexpression of let-7 rescues the premature myogenic differentiation phenotype caused by H19 knockdown, confirming that H19 affects myogenesis at least in part by antagonizing the function of the let-7 family of miRNAs. Consistent with this view, two of the well-documented targets of let-7, HMGA2 and IGF2, have also been found to be downstream effectors of the H19 lncRNA during muscle differentiation [164]. Intriguingly, two conserved miRNAs (miR-675-3p and miR-675-5p) that play important roles during myogenic differentiation were recently found to reside in the first exon of the H19 lncRNA, thus suggesting that H19 lncRNA also serves as a primary miRNA transcript [60]. It would be interesting to further study how H19 achieves both functions of miRNA sponge and miRNA precursor during myogenesis and discover which function is more critical or important* in vivo*.

Another excellent example of lncRNAs acting as molecular decoys is linc-MD1, the first muscle-specific lncRNA identified by Gabellini and colleagues.* Linc-MD1* is encoded by a genomic locus that overlaps with the bicistronic miR-206 and miR-133b transcript-coding region [165]. Linc-MD1 is required for appropriate muscle differentiation, at least in part because it regulates the levels of Myocyte Enhancer Factor 2C (MEF2C) and Mastermind-like protein 1 (MAML1), via the mechanism of sponging endogenous miR-133 and miR-135 in cytoplasm [165]. Similar to H19, linc-MD1 can also serve as a pre-miRNA transcript, as it encodes miR-133b [165]. Interestingly, HuR, an RNA binding protein that stabilizes the mRNAs of several myogenic factors during muscle differentiation, is a target of miR-133. HuR facilitates the formation of the linc-MD1-miRNA complex and its accumulation in cytoplasm, thus promoting linc-MD1's miRNA sponge activity [166]. Therefore, there is a delicate and complex regulatory circuit between linc-MD1, miR-133, and HuR, which is critical for appropriate muscle differentiation. Lastly, the substantial downregulation of linc-MD1 in primary myoblasts of patients with DMD suggests that it is likely involved in the pathogenesis of this muscle disorder [165].

Amazingly, about one-third of lncRNAs are found to contain at least one short interspersed element (SINE) sequence, suggesting that they may actively participate in the Staufen 1- and Staufen 2- (STAU1- and STAU2-) mediated mRNA decay (SMD) process [167, 168]. When a SINE within the 3′ UTR of a protein-coding RNA forms intermolecular base pairing with a partially complementary SINE of one or more lncRNAs, the resulting double-stranded RNA (dsRNA) can be recognized by Staufen 1 and Staufen 2 and degraded through SMD [169, 170]. Several mRNAs that encode proteins which play important roles during myogenesis have SINE sequences in their 3′ UTRs (two recently reported examples are Cdc6 and Traf6) and thus can be bound by SINE-containing lncRNAs and targeted for degradation through SMD, inhibiting their translation and thus their functions during myogenesis [170].

### 3.4. lncRNAs as Subcellular Domain Scaffold to Regulate Muscle Differentiation

The lncRNA Malat1 is abundantly expressed in various types of cells and regulates their proliferation and metastasis [171, 172]. Malat1 is predominantly located in nuclear speckles and regulates both gene transcription and pre-mRNA splicing [173, 174]. Through interaction with Cbx4, Malat1 regulates gene expression by modulating chromatin translocation among nuclear domains [174], whereas it regulates alternative splicing of certain pre-mRNAs through interaction with the SR family of splicing factors [173]. During muscle differentiation, Malat1 is upregulated, and it has been identified as a downstream target of myostatin, a well-known negative regulator of myogenesis [175]. Knockdown of Malat1 suppresses myoblast proliferation and differentiation [176]. However, the exact mechanism underlying this observation remains elusive and needs further investigation.

### 3.5. lncRNAs and Duchenne Muscular Dystrophy (DMD)

Dystrophin is the causative gene of DMD; however, the molecular mechanisms and cellular events underlying DMD pathophysiology are still not fully understood. A recent study using tiling arrays has identified about 14 new lncRNAs that originate from the dystrophin gene, which consists of 79 exons, making it the largest human gene. Some of these lncRNAs appear to target the promoters of the dystrophin gene* in trans* to repress the expression of some dystrophin isoforms [177]. However, it remains to be determined how these lncRNAs contribute to the pathophysiology of the dystrophic muscle. In addition, in DMD patients with mental retardation, a specific chromosomal inversion event generates an lncRNA, KUCG1, which may account for the clinical mental retardation symptoms [178]. Furthermore, as mentioned above, the level of linc-MD1 is greatly reduced in the muscle of DMD patients, and linc-MD1 overexpression can rescue the defective myogenic differentiation and restore the normal expression of* Maml1, Mef2c, Myog and Mhc* [165]. Therefore, enhancing linc-MD1 levels in DMD patients promises to be a potential therapeutic strategy.

### 3.6. Facioscapulohumeral Muscular Dystrophy (FSHD), an lncRNA Link

Facioscapulohumeral muscular dystrophy (FSHD) is an autosomal-dominant hereditary muscle disease that causes progressive weakness and loss of skeletal muscles [179]. As the third most common muscular dystrophy, it affects 1 in 14,000 people [179]. The genetic region involved in FSHD does not encode any protein but rather contains a 3.3 kb macrosatellite* D4Z4* repeat [180]. While normal people have anywhere from 11 to more than 100 copies of* D4Z4* repeats in the subtelomeric region of chromosome 4q35, FSHD patients tend to have less than ten repeats [181–183]. A recently identified chromatin-associated lncRNA, DBE-T, is selectively produced in FSHD patients and is associated with the derepression of genes in the 4q35 locus [184]. Mechanistically, DBE-T directly interacts with and recruits the Trithorax group protein Ash1L, a histone methyltransferase, to the FSHD locus. Locally increasing Ash1L results in histone H3K36 dimethylation, which in turn relaxes the local chromatin, resulting in the activation of DBE-T as well as neighboring genes at the FSHD loci [184]. Therefore, a feedforward mechanism involving the lncRNA DBE-T and Ash1L in FSHD patients promotes the derepression of chromosomal 4q35 region and thus contributes to the pathogenesis of FSHD.

## 4. Perspectives

The discovery of noncoding RNAs (miRNAs and lncRNAs) has dramatically expanded our understanding of how gene expression is regulated. miRNAs, which regulate gene expression by targeting protein-coding genes, add a posttranscriptional regulatory mechanism in gene regulation. With the increasing number of publications regarding miRNA function in skeletal muscles, it has become clear that miRNAs are an integral part of myogenic regulatory networks. Although much progress has been made in identifying and verifying targets of specific miRNAs in skeletal muscle cells and in elucidating the functional mechanism of many miRNAs in skeletal muscle biology, the relationship between miRNAs and various muscle disorders has yet to be fully understood. In addition, how to harness these small RNAs to develop effective and economic diagnostic and therapeutic tools is still a question to be addressed. Finally, the function of miRNAs in skeletal muscle metabolism appears to be one of the understudied areas of miRNA biology. Skeletal muscle is a metabolically active tissue, and it would be of great interest to define whether miRNAs are also an integral part of the metabolic regulatory network in muscle. Undoubtedly, future work about the molecular mechanisms of miRNAs in skeletal muscle diseases and their therapeutic applications will emerge and contribute to a comprehensive understanding of miRNA biology in skeletal muscles.

Unlike miRNAs, lncRNAs are identified only recently and we are still at the “infant stage” of studying this novel class of noncoding RNAs in skeletal muscle. Overall, as for miRNAs, there is emerging evidence that lncRNAs are important regulators of muscle biology. It is likely that there are still many muscle-related lncRNAs to be discovered and that they will act via multiple distinct mechanisms, some of which may be unprecedented. Future studies will also need to examine whether aberrant lncRNAs are linked with various muscle diseases and what their roles are in pathogenesis. In addition, given the RNA nature of lncRNAs and miRNAs, it is intriguing to speculate that lncRNAs and miRNAs can coordinately regulate the expression of certain mRNAs. To some extent, the fact that lncRNAs can reduce the levels of miRNAs by functioning as their sponges supports this hypothesis. Furthermore, in the light of the recently identified muscle-specific micropeptide myoregulin, encoded by a putative noncoding RNA [185], we cannot rule out the possibility that some “noncoding” lncRNAs might actually encode physiological active micropeptides. In conclusion, we are at an exciting time in noncoding RNA biology and in the study of their functions in skeletal muscle biology and muscle diseases. We are confident that more and more noncoding RNA-based diagnostic and therapeutic applications will emerge in the future.

## Figures and Tables

**Table 1:** miRNAs and targets implicated in the development of skeletal muscle.

miRNA	miRNA target(s)	Function in myogenic differentiation	References
miR-1	HDAC4 Pax7	Promote differentiation Inhibit proliferation	[26, 33]
miR-133	SRF UCP2	Promote proliferation Promote differentiation	[26, 110]
miR-206	Pol*α*1, Cx43; Pax3/7, Fstl1, and Utrn; Notch3 and Igfbp5	Promote differentiation Inhibit proliferation	[33–35, 41, 63, 64, 111]
miR-23a	Myh	Inhibit differentiation	[112]
miR-24	Smad 3	Promote differentiation	[59]
miR-26a	Ezh2 Smad 1 and Smad 4	Promote differentiation	[48, 49]
miR-27	Pax3 Myostatin	Promote satellite cell activation Promote differentiation	[37, 57, 58]
miR-29	HDAC4, YY1, Ring1, Rybp, Akt3, Col, and Lims1	Promote differentiation, inhibit proliferation, and fibrosis	[50–52, 58, 113, 114]
miR-31	Myf5	Maintenance of quiescence/stemness	[39]
miR-124	Dlx5	Inhibit differentiation	[115]
miR-125b	IGF-II	Inhibit differentiation	[116]
miR-128a	Insr; IRS1 and Pik3r1	Inhibit proliferation	[117]
miR-148a	ROCK1	Promote differentiation	[118]
miR-146b	Smad4, Notch1, and Hmga2	Promote differentiation	[119]
miR-155	MEF2A	Inhibit differentiation	[120]
miR-181	Hox-A11	Promote differentiation	[32]
miR-186	Myogenin	Inhibit differentiation	[31]
miR-195/497	Cdc25, Ccnd	Maintenance of quiescence/stemness	[43]
miR-199a-3p	IGF-1, mTOR, and RPS6KA6	Inhibit differentiation	[121]
miR-199a-5p	FZD4, JAG1, and WNT2	Promote proliferation	[99]
miR-203 miR-203b	c-JUN, MEF2C, and MyoD	Inhibit proliferation and differentiation	[122, 123]
miR-208b miR-499	Sox6, Pur*β*, Sp3, and HP-1*β*	Fiber type determination	[67]
miR-214	Ezh2	Promote differentiation	[47, 124]
miR-221/222	p27, MyoD	Inhibit differentiation	[125, 126]
miR-322/424	Cdc25A	Promote differentiation	[44]
miR-351	E2f3	Promote proliferation	[127]
miR-378	MyoR	Promote differentiation	[128]
miR-486	Pax7	Promote differentiation	[38]
miR-489	Dek	Maintenance of quiescence/stemness	[129]
miR-503	Cdc25A	Inhibit proliferation	[44]
miR-669	MyoD	Inhibit proliferation	[130]
miR-675	Smad1, Smad5, and Cdc6	Promote differentiation	[60]
miR-682	Unknown	Promote proliferation	[131]
miR-1192	HMGB1	Inhibit differentiation	[132]
miR-3906	Homer-1b	Promote differentiation	[133]
